# Apolipoprotein B in the Risk Assessment, Diagnosis, and Treatment of Cardiometabolic Diseases

**DOI:** 10.26502/fccm.92920466

**Published:** 2025-09-04

**Authors:** Shaan Patel, Hina Patel, Shaanali Mukadam, Devendra K Agrawal

**Affiliations:** Department of Translational Research, College of Osteopathic Medicine of the Pacific, Western University of Health Sciences, Pomona, California 91766 USA

**Keywords:** Apolipoprotein B, Atherosclerosis, Cardiovascular disease, Dyslipidemia, GLP-1 receptor agonist, Hyperglycemia, Hyperlipidemia, Insulin resistance, Low-density lipoprotein, Metformin, PCSK9 inhibitor, SGLT2 inhibitor

## Abstract

Apolipoprotein B (ApoB) has emerged as a central biomarker and mechanistic driver of atherosclerotic cardiovascular disease (ASCVD), outperforming traditional lipid metrics in both risk stratification and therapeutic targeting. In this article a critical evaluation of the information is presented on the molecular biology, metabolic regulation, and clinical relevance of ApoB isoforms, ApoB100 and ApoB48, which play their own distinct, yet complementary roles in hepatic and intestinal lipid transport. The ways in which ApoB particle density is influenced by insulin resistance, nutrient status, hepatic lipid flux, inflammation, and genetic variation, all of which contribute to dyslipoproteinemic phenotypes associated with ASCVD and metabolic syndrome. Importantly, ApoB levels provide a direct measure atherogenic particle number, offering superior predictive value over low-density lipoprotein cholesterol (LDL-C), particularly in cases of lipid discordance and among statin-treated patients with residual cardiovascular risk. Emerging evidence demonstrates therapies targeting ApoB reduction, including statins, PCSK9 inhibitors, and glucose-lowering agents such as GLP-1 receptor agonists, can significantly reduce major adverse cardiovascular events. However, the lipid-modulating effects of agents like SGLT2 inhibitors, metformin, and thiazolidinediones are variable or independent of ApoB changes. The classification of four ApoB-related dyslipoproteinemic phenotypes, normotriglyceridemic hyperApoB, hypertriglyceridemic normoApoB, hypertriglyceridemic hyperApoB, and hyperchylomicronemia, offers a more nuanced approach to cardiovascular risk assessment than LDL-c alone. Collectively, these findings support the integration of ApoB measurement into routine clinical practice as both diagnostic tool and therapeutic target, with the potential to substantially enhance personalized management of cardiometabolic disease.

## Introduction

Cardiovascular disease (CVD) remains the leading cause of mortality globally, responsible for approximately 20.5 million deaths each year [1]. A central mechanism underlying the development of CVD is atherosclerosis, a progressive pathological process marked by the accumulation of lipid-rich plaques within the arterial intima [2–6]. Genetics, epigenetics, and environmental factors play a significant role in the underlying pathogenesis of cardiovascular diseases [7–9]. Lipoproteins, which facilitate the transport of lipids through the circulatory system, are critically involved in this process, with dysregulation in their composition and function being strongly linked to the onset and progression of atherosclerotic cardiovascular disease (ASCVD). Within these lipoprotein particles, Apolipoproteins serve essential roles in lipid metabolism, cholesterol trafficking, and the initiation and progression of plaque formation [10].

Apolipoproteins are integral protein components of lipoproteins, serving to stabilize lipid particles and mediate the transport of cholesterol, triglycerides, and phospholipids through the circulatory system. Among them, Apolipoprotein (Apo) A and ApoB have been most extensively studied for their roles in cardiovascular physiology and pathology [11,12]. ApoA constitutes the primary structural protein of high-density lipoprotein (HDL), often termed “good cholesterol,” owing to its critical function in reverse cholesterol transport, the process by which surplus cholesterol is mobilized from peripheral tissues and delivered to the liver for excretion [12]. In contrast, ApoB is the principal structural protein of atherogenic lipoproteins, including low-density lipoprotein (LDL), intermediate-density lipoprotein (IDL), and very-low-density lipoprotein (VLDL) [13]. Through its role in cholesterol delivery to peripheral tissues, ApoB is closely associated with the pathogenesis of atherosclerosis and plaque formation [14–20]. Co-morbidities, such as metabolic syndrome and infections, accelerate inflammatory response and develop vulnerable plaques leading to myocardial infarction, stroke and other thrombotic complications [21–30].

ApoB exists in two isoforms: ApoB100 and ApoB48, which differ in their structure, site of synthesis, and physiological function. ApoB100, synthesized in the liver, is essential for the binding of LDL to its receptor, thereby facilitating the systemic transport of cholesterol [31]. ApoB48, produced in the intestine, is critical for the assembly of chylomicrons, which mediate the transport of dietary lipids [32]. The structural and functional divergence between these isoforms highlights their distinct yet complementary roles in lipid metabolism. Their relevance extends beyond atherosclerosis to encompass a range of lipid-associated disorders, including familial hypercholesterolemia, non-alcoholic fatty liver disease, and type 2 diabetes, positioning them as central targets in the prevention and management of cardiometabolic diseases [33].

Recent advancements in molecular biology and clinical research have highlighted the significance of Apolipoprotein concentrations as robust biomarkers for assessing cardiovascular disease (CVD) risk. In particular, the Apolipoprotein B to Apolipoprotein A (ApoB/ApoA) ratio has emerged as a superior predictor of cardiovascular events compared to conventional lipid metrics such as LDL-C [34,35]. This ratio reflects the balance between atherogenic and anti-atherogenic lipoproteins, offering a more accurate assessment of lipid-related cardiovascular risk [36]. Elucidating the molecular mechanisms by which distinct Apolipoproteins modulate lipid metabolism and contribute to CVD pathogenesis is essential for the development of precision therapies aimed at reducing cardiovascular morbidity and mortality.

## Differential Expression and Roles of ApoB Isoforms

ApoB plays a pivotal role in the metabolism of atherogenic lipoproteins, and both its overproduction and deficiency are associated with a variety of metabolic and cardiovascular pathologies [37]. Consequently, investigating the mechanisms that regulate ApoB homeostasis is important for understanding its role in disease pathogenesis and identifying therapeutic targets.

The two physiological forms of ApoB, ApoB100 and ApoB48, are encoded by a single *APOB* gene, located on chromosome 2 [38]. Despite being derived from the same gene, these isoforms are expressed in a tissue specific manner: ApoB100 is produced in the liver, while ApoB48 is produced in the small intestine. Hepatic ApoB100 is 4536 amino acids in length and has a molecular mass of approximately 550 kDa. It plays a critical role in the assembly and receptor-mediated clearance of VLDL, IDL, and LDL particles, serving as the key ligand for the hepatic LDL receptor. ApoB100 has a large, globular N-terminal domain and C-terminal domain called the B-belt. The amphipathic B-sheet within the C-terminal domain contains nine interstrand inserts that wrap around the LDL particle and provide structural support and proper presentation of the particle to the LDL receptor-binding domain for clearance [39].

While ApoB100 mediates systemic cholesterol transport and LDL clearance, the intestinal isoform ApoB48 performs a distinct but complementary role in lipid metabolism. ApoB48 consists of 2512 amino acids and has a molecular mass that is approximately 48% of that of ApoB100 [40]. This truncated protein isoform arises from a highly specific post-transcriptional RNA editing mechanism in intestinal cells, in which a cytidine in the ApoB mRNA is deaminated to uridine by a process catalyzed by the enzyme APOBEC1 (Apolipoprotein B mRNA editing enzyme, catalytic polypeptide 1) [40]. This editing event introduces a premature stop codon, resulting in the translation of a truncated protein that is half of the molecular weight of the native ApoB100 isoform and lacks the C-terminal LDL receptor-binding domain. As a result, ApoB48 cannot facilitate LDL receptor binding. Instead, it is essential in the assembly of chylomicrons, which transport dietary triglycerides and cholesterol via the lymphatic system to peripheral tissues [41].

Nutritional status, particularly dietary fat intake and insulin sensitivity, significantly influences ApoB mRNA stability and translation [42]. In a nutrient-rich state, particularly with high carbohydrate and fat availability, hepatic translation of ApoB100 is upregulated, contributing to increased VLDL production [42]. A key determinant of the fate of ApoB during translation is the availability and activity of microsomal triglyceride transfer protein (MTP) [43]. MTP is essential for the lipidation of nascent ApoB, a process required for its proper folding and assembly into lipoprotein particles. In the absence of sufficient lipid transfer by MTP, the nascent ApoB polypeptide fails to attain a stable conformation and is targeted for proteasomal degradation [44]. This process, known as endoplasmic reticulum-associated degradation (ERAD), ensures that misfolded or excess ApoB is eliminated before it can accumulate and contribute to lipotoxicity or inefficient lipoprotein secretion [45]. Together, these regulatory mechanisms tightly control ApoB availability and secretion, linking nutrient status to lipoprotein metabolism and the maintenance of lipid homeostasis.

## Pathophysiological Determinants of ApoB Particle Density

Because each ApoB-containing lipoprotein particle (e.g., VLDL, LDL, and their remnants) contains a single molecule of ApoB, plasma ApoB concentration directly reflects the number of circulating atherogenic particles. Hepatic VLDL production is a principal determinant of this pool. Each nascent VLDL particle assembles around an ApoB100 scaffold and is lipidated with TG through microsomal transfer. The efficiency of this assembly and the number of particles secreted depend on lipid availability and hepatocellular signaling. Insulin serves as a critical physiological regulator, acutely reducing VLDL-ApoB output by promoting intracellular degradation of ApoB and limiting lipidation of nascent particles [46]. This postprandial insulin response restrains excessive particle release. Conversely, disruption of key molecular components involved in VLDL biogenesis can suppress ApoB secretion. For example, hepatocyte-specific deletion of TIAL1 (Tia1 cytotoxic granule associated RNA binding protein like 1) in mice reduces ApoB synthesis by approximately 50%, resulting in a marked decline in VLDL particle output [47].

Insulin resistance (IR) and related metabolic abnormalities drive a pathological increase in ApoB particle number. In the insulin-resistant liver, hyperinsulinemia fails to suppress VLDL production, while elevated free fatty acid flux from insulin-resistant adipose tissue enhances hepatic triglyceride (TG) synthesis. This promotes overproduction of large, TG-rich VLDL1 particles [46]. Kinetic studies in humans with visceral obesity confirm significantly increased VLDL-ApoB secretion rates in insulin-resistant individuals [46]. The presence of hepatic steatosis, non-alcoholic fatty liver disease (NAFLD), further amplifies this effect by increasing the lipid substrate available for VLDL assembly [48]. Excess VLDL1 production ultimately leads to increased generation of LDL particles via progressive lipolysis and remodeling. These LDL particles are typically small and cholesterol-poor, a hallmark of IR-associated dyslipidemia [48]. Improvements in insulin sensitivity can reverse these abnormalities. After substantial weight loss, VLDL1-ApoB secretion declines, and clearance of TG-rich remnants improves in parallel with reductions in IR [46], highlighting the tight link between metabolic status and ApoB particle dynamics.

Genetic factors also shape ApoB particle concentration by modifying both lipoprotein production and clearance. Variants that impair hepatic VLDL assembly result in reduced ApoB secretion. An example of this is the TM6SF2 E167K variant, which impairs VLDL export and predisposes to hepatic fat accumulation. Carriers of this variant secrete fewer ApoB-containing particles, lowering plasma ApoB levels while promoting intracellular TG retention [48]. In contrast, genetic defects that impair lipoprotein catabolism increase ApoB levels by limiting clearance. Familial hypercholesterolemia due to mutations in the LDL receptor or PCSK9 illustrates this mechanism, while loss-of-function variants that enhance lipoprotein clearance confer protection. Individuals with inactivating mutations in angiopoietin-like protein 3 (ANGPTL3), an endogenous inhibitor of lipoprotein lipase, exhibit lifelong reductions in LDL and VLDL concentrations [49]. Mechanistic studies suggest that ANGPTL3 inhibition accelerates VLDL lipolysis, yielding remnant particles that are rapidly cleared before conversion to LDL [4]. In sum, genetic alterations that enhance hepatic output or reduce clearance raise ApoB particle density, while those that blunt production or augment clearance have the opposite effect [49].

Systemic inflammation modulates ApoB lipoprotein profiles by disrupting both production and clearance pathways. Acute inflammatory responses, driven by cytokines such as IL-1β, IL-6, and TNF-α, promote hypertriglyceridemia by stimulating hepatic VLDL synthesis and inhibiting lipoprotein lipase-mediated TG clearance [50]. This leads to transient increases in circulating ApoB particle levels while chronic inflammation often suppresses ApoB and cholesterol levels by sustaining cytokine-induced upregulation of catabolic pathways [50]. This is exemplified in rheumatoid arthritis (RA), where active inflammation correlates with abnormally low LDL-ApoB levels. Notably, treatment with the IL-6 receptor antagonist tocilizumab in RA patients raises LDL levels by approximately 20% within months [50], indicating a reversal of inflammation-induced suppression of lipoprotein production. These observations highlight the dynamic influence of inflammatory signaling on hepatic lipoprotein output and peripheral clearance, with important implications for ApoB particle burden in disease states.

## Pathophysiological Characteristics of the 4 Major Dyslipoproteinemic Phenotypes

A comprehensive understanding of lipid metabolism of both normal and pathological phenotypes is essential for accurate cardiovascular risk stratification and the development of targeted therapeutic strategies. The normal lipoprotein phenotype is characterized by efficient production and clearance of VLDL and LDL particles, typically reflected by ApoB levels below 105 mg/dL, triglyceride concentrations near or below 130 mg/dL, and a triglyceride-to-ApoB ratio less than 10:1. This profile indicates a balanced lipoprotein metabolism and is associated with low atherogenic potential [51]. In contrast, four principal dyslipoproteinemic phenotypes, primarily involving disturbances in ApoB-containing lipoproteins, have been identified as major contributors to ASCVD and other lipid-related disorders [52]. These phenotypes are defined by varying levels of ApoB, triglycerides, and alterations in lipoprotein particle composition, reflecting distinct metabolic disturbances not captured by conventional lipid measures such as LDL-C alone [53].

The first phenotype, normotriglyceridemic hyperApoB, is marked by elevated ApoB concentrations despite normal triglyceride levels [51]. This profile reflects an increased number of small, dense LDL particles, which are particularly atherogenic due to their enhanced ability to penetrate the arterial intima and undergo oxidative modification. Despite appearing normolipidemic on standard lipid panels, individuals with this phenotype exhibit significantly elevated ASCVD risk due to an excess of atherogenic particles [54].

In contrast, the hypertriglyceridemic normoApoB phenotype presents with elevated triglycerides but normal ApoB levels, indicating that the increase in circulating lipids is driven by triglyceride enrichment per particle rather than particle number [55]. This phenotype is characterized by the accumulation of large, triglyceride-rich VLDL particles, often associated with insulin resistance and hepatic steatosis [56]. Although the ASCVD risk is relatively modest, patients are more prone to hypertriglyceridemia-induced pancreatitis, particularly when triglyceride levels exceed 500–1000 mg/dL [57].

The hypertriglyceridemic hyperApoB phenotype represents a more atherogenic profile, with elevations in both ApoB and triglycerides. This pattern indicates a dual burden of increased particle number and increased triglyceride content, encompassing both small, dense LDL and remnant VLDL particles [51]. Frequently associated with metabolic syndrome and type 2 diabetes mellitus, this phenotype is pathophysiologically linked to hepatic overproduction of ApoB-containing lipoproteins, delayed lipolysis, and increased cholesteryl ester transfer protein (CETP)-mediated lipid exchange [21,58]. It confers a high risk of ASCVD due to the cumulative effects of remnant cholesterol and particle-mediated vascular injury.

Lastly, the hyperchylomicronemic phenotype is defined by extreme hypertriglyceridemia due to impaired clearance of chylomicrons, typically caused by genetic defects in lipoprotein lipase (LPL) or its co-factors, such as ApoC-II and GPIHBP1 [59]. This contributes to atherosclerosis through endothelial cell inflammation, which also poses a significant risk for acute pancreatitis [60]. This phenotype may be exacerbated by secondary factors such as uncontrolled diabetes, alcohol intake, or certain medications.

Collectively, these dyslipoproteinemic phenotypes highlight the complexity of lipid metabolism and underscore the limitations of LDL-C as a solitary marker for cardiovascular risk. A more nuanced understanding of ApoB particle dynamics and triglyceride metabolism is critical for identifying residual risk and informing personalized lipid-lowering strategies.

## Superiority of ApoB as a Biomarker in Diagnosis and Treatment

ApoB has emerged as a superior biomarker for atherosclerotic cardiovascular risk assessment compared to traditional cholesterol measures. ApoB is the primary protein of all atherogenic lipoproteins, including LDL, VLDL, IDL, and lipoprotein(a). Because each of these particles carries exactly one ApoB molecule, measuring ApoB provides a direct count of the total number of circulating atherogenic particles [61]. This is clinically significant because atherosclerosis is driven by the accumulation of ApoB-containing lipoproteins in the arterial wall, where the cholesterol carried by these particles is deposited after the particles are trapped, inciting plaque formation and inflammation [61]. In contrast, LDL-cholesterol (LDL-C) quantifies the cholesterol content within LDL particles, which varies substantially between particles. For example, a patient with many small, cholesterol-depleted LDL particles may have a normal LDL-C level but an elevated ApoB, indicating an abundance of LDL particles with higher risk that LDL-C alone might underestimate. Conversely, a patient with a few large, cholesterol-rich LDL particles could have high LDL-C but a normal ApoB count, and their risk may be lower than LDL-C suggests. By directly counting particles, ApoB captures such discordance and more accurately reflects the true atherogenic burden [62].

Multiple contemporary studies have confirmed that ApoB outperforms LDL-C (and other lipid markers) in predicting cardiovascular events. In a recent analysis combining a large primary-prevention cohort and secondary-prevention trial populations, ApoB was the only lipid parameter that remained independently associated with myocardial infarction (MI) risk when controlling for LDL-C and triglycerides [63]. In that study, which included over 389,000 individuals from the UK Biobank and approximately 40,000 patients with established coronary disease, LDL-C and non-HDL cholesterol lost significance once ApoB was considered, while ApoB retained a strong association with future MI [63]. When ApoB, LDL-C, and other lipids were assessed concurrently, only ApoB consistently predicted outcomes, highlighting that myocardial infarction risk was most accurately captured by the number of ApoB-containing particles [63]. Similarly, a 2024 cohort study introduced the concept of “excess ApoB”, the ApoB level above what would be expected from a given LDL-C and showed a dose-dependent increase in ASCVD events with higher excess ApoB [64]. Individuals with ApoB levels substantially exceeding those predicted by their LDL-C experienced markedly higher rates of MI and ASCVD, reinforcing that ApoB offers predictive insight beyond cholesterol mass across the full LDL-C range [64]. The association was consistent in both women and men, which highlights the broad applicability of ApoB as a risk marker [64].

The clinical relevance of this distinction becomes even more apparent in situations of lipid metric discordance and in patients receiving lipid-lowering therapy. In statin-treated patients, an elevated ApoB can identify “residual risk” even when LDL-C is at goal. In 2021, Johannesen et al. examined over 13,000 statin-treated individuals and found that those with high ApoB, but relatively low LDL-C had significantly higher risk of myocardial infarction and all-cause mortality, while patients with high LDL-C but low ApoB did not have excess risk [62]. In quantitative terms, a discordant profile of high ApoB (above median) despite LDL-C below median was associated with approximately 1.5-fold increase in risk of MI, while an isolated high LDL-C (with normal ApoB) conferred no significant risk elevation [62]. Such discordance analyses reinforce the superiority of ApoB over LDL-C as a marker of residual risk in statin-treated patients [62]. Non-HDL cholesterol also outperformed LDL-C in these analyses, but ApoB showed the greatest precision in risk stratification [62]. The investigators concluded that in patients on lipid-lowering therapy, elevated ApoB and non-HDL-C, but not LDL-C, correlate with residual cardiovascular risk, solidifying the role of ApoB as the superior indicator of whether a patient remains at risk [62]. Measuring ApoB can uncover high-risk patients whose LDL-C appears controlled while also avoiding overestimation of risk in those with high LDL-C but low particle count.

Further supporting its clinical utility, ApoB also emerges as the most effective target for lipid-lowering therapy. Evidence from clinical trials supports the concept that “lower is better” for ApoB. In a post-hoc analysis of the ODYSSEY outcomes trial which tested alirocumab in post-acute coronary syndrome patients on statins, patients who achieved very low ApoB levels had the fewest recurrent events, even if their LDL-C was already aggressively lowered [65]. Specifically, the incidence of major adverse cardiac events (MACE) in the alirocumab-treated group declined monotonically as achieved ApoB fell, with the lowest rates observed in patients reaching ApoB ≤35 mg/dL [65]. Perhaps most telling, achieved ApoB was a stronger predictor of outcomes than achieved LDL-C or non-HDL-C. When adjusted for ApoB, differences in LDL-C or non-HDL-C no longer correlated with risk, but when adjusting for LDL-C, ApoB continued to predict events [65]. These findings imply that therapies targeting maximal reduction in ApoB, reflecting the number of circulating atherogenic particles, may offer the most robust protection against future cardiovascular events. They also raise the possibility that treatment guidelines could refine targets to include ApoB levels, for instance by aiming for ApoB <35 mg/dL in very high-risk individuals, to ensure all residual risk is addressed [65].

Collectively, recent evidence supports ApoB as a more accurate and clinically actionable biomarker for atherosclerotic cardiovascular disease. It more accurately stratifies risk than LDL-C because it reflects the true particle burden driving plaque formation [63,64]. It also provides incremental prognostic information during treatment, correlating with outcomes more closely than traditional cholesterol metrics [5]. ApoB can be measured directly, inexpensively, and without fasting, with greater analytical precision than calculated LDL-C [61]. Indeed, modeling estimates suggest that replacing LDL-C with ApoB as the primary treatment target could prevent substantially more cardiovascular events, on the order of hundreds of thousands over a decade in the U.S. population [61]. Embracing ApoB for both risk assessment and therapeutic guidance could improve the identification of high-risk patients and enable more effective atherogenic particle reduction, ultimately translating to better cardiovascular outcomes [61].

## Emerging Treatments

In individuals with type 2 diabetes mellitus (T2DM), ApoB levels are frequently elevated due to the characteristic atherogenic dyslipidemia associated with insulin resistance. This dyslipidemic profile is characterized by elevated triglycerides, reduced HDL-C, and an increased proportion of small, dense LDL particles [66]. These small, dense LDL particles are more atherogenic than larger LDL particles because they are more susceptible to oxidative modification, have a greater propensity to penetrate the arterial wall, and exhibit prolonged plasma residence time [67]. Each atherogenic lipoprotein particle, including LDL, VLDL, IDL, and lipoprotein(a), contains one molecule of ApoB-100, making ApoB a direct measure of the number of circulating atherogenic particles. Consequently, patients with T2DM often exhibit a discordance between LDL-C and ApoB concentrations, wherein LDL-C levels may appear within target ranges while ApoB levels remain elevated, reflecting a high number of cholesterol-depleted yet atherogenic particles [68]. This discordance underscores the importance of measuring ApoB to accurately assess cardiovascular risk in T2DM patients.

Among glucose-lowering therapies, glucagon-like peptide-1 receptor agonists (GLP-1 RAs), such as liraglutide and semaglutide, have demonstrated modest but consistent reductions in ApoB concentrations, an effect likely mediated through weight loss, improved insulin sensitivity, and reductions in hepatic VLDL production [22,69]. Beyond lipid modulation, GLP-1 RAs have been shown to reduce MACE in high-risk patients with T2DM, as evidenced in large-scale randomized controlled trials such as LEADER and SUSTAIN-6 [70]. The reduction in ApoB, although modest, may contribute to these cardiovascular benefits by decreasing the number of circulating atherogenic lipoprotein particles, including small dense LDL and VLDL remnants, both of which are elevated in insulin-resistant states.

Sodium-glucose cotransporter-2 (SGLT2) inhibitors, including empagliflozin and dapagliflozin, exhibit largely neutral effects on ApoB, despite modest increases in LDL cholesterol observed in some studies [71]. This paradoxical lipid profile may reflect a shift toward larger, less atherogenic LDL particles without a corresponding increase in ApoB, suggesting no increase in the total number of atherogenic particles. However, findings across studies remain heterogeneous, as several trials have reported no significant changes in LDL cholesterol or triglycerides, indicating that lipid effects may vary based on population characteristics, background therapy, and study duration [72,73]. Despite minimal direct impact on ApoB, SGLT2 inhibitors have consistently demonstrated robust reductions in cardiovascular and renal outcomes, particularly heart failure hospitalization and progression of chronic kidney disease, effects believed to be driven by mechanisms independent of lipid modification [74].

Metformin, the first-line pharmacologic agent for T2DM, has only minimal or inconsistent effects on ApoB concentrations, with most studies showing either no change or modest reductions in the context of improved glycemic control and weight loss [75]. Thiazolidinediones (e.g., pioglitazone), which enhance insulin sensitivity through peroxisome proliferator-activated receptor gamma (PPAR-γ) activation, also show variable effects on ApoB levels [76]. In some populations, particularly those with high baseline triglycerides or insulin resistance, pioglitazone may reduce ApoB and atherogenic lipoprotein particle concentrations [77]. In other cases, however, increases in LDL cholesterol and weight gain may offset these benefits. Nevertheless, pioglitazone has demonstrated cardiovascular benefit in secondary prevention settings, particularly in patients with prior stroke or extensive insulin resistance, suggesting the involvement of lipid-independent mechanisms, such as improved adipose tissue function and anti-inflammatory effects [78].

Lipid-lowering therapies exert more pronounced effects on ApoB levels. Statins remain the most effective agents, consistently reducing ApoB by approximately 20–40% through upregulation of LDL receptors [79]. PCSK9 inhibitors, such as evolocumab and alirocumab, offer even greater ApoB reductions, often exceeding 40%, and are typically reserved for patients at very high cardiovascular risk [80]. Ezetimibe provides an additional 10–15% reduction in ApoB when used in combination with statins [81]. Fibrates modestly reduce ApoB, particularly in individuals with elevated triglycerides [82], while high-dose purified EPA formulations (e.g., icosapent ethyl) may also yield modest reductions, though their cardiovascular benefits may not be entirely mediated through ApoB lowering [83]. In contrast, sulfonylureas and exogenous insulin can potentially increase ApoB levels, particularly in the context of weight gain or worsening insulin resistance [84].

These findings underscore that while some glucose-lowering therapies may modestly influence ApoB and related lipoprotein profiles, the cardiovascular and metabolic benefits of these agents often extend beyond their effects on traditional lipid markers. Therefore, ApoB measurement may serve as a useful adjunct in risk stratification but should be interpreted within the broader context of each agent’s pleiotropic effects.

## Treatment Questions

The effects of glucose-lowering therapies on ApoB and atherogenic lipoproteins prompt several important clinical and research inquiries. In the case of GLP-1 RAs, it remains to be clarified how much the modest reductions in ApoB contribute to the cardiovascular benefits observed in patients with T2DM. Additionally, it is worth exploring whether certain subpopulations, such as those with obesity, elevated triglycerides, or hepatic steatosis, experience more pronounced improvements in ApoB and related lipoproteins. This raises the possibility that ApoB measurement could serve as a useful tool in guiding treatment intensification with GLP-1 RAs, especially among patients who have reached LDL cholesterol targets but remain at residual risk [85].

Regarding SGLT2 inhibitors, their neutral effects on ApoB, despite clear cardiovascular and renal benefits, suggest that these outcomes may be largely independent of changes in atherogenic lipoprotein concentrations. However, it remains unclear whether SGLT2 inhibitors alter the qualitative aspects of LDL and VLDL particles, such as size and density, which may not be reflected in ApoB levels alone [86]. Further investigation is needed to determine whether patients with elevated ApoB but preserved kidney function derive similar cardiovascular protection from SGLT2 inhibition compared to those with lower ApoB levels.

Metformin, widely regarded as the cornerstone of T2DM treatment, appears to have minimal and inconsistent effects on ApoB concentrations. This raises questions about the sufficiency of metformin’s lipid effects in patients with significant atherogenic lipoprotein burdens and whether combination with lipid-targeting agents is warranted. Moreover, the potential for metformin to influence ApoB metabolism or hepatic lipoprotein secretion in insulin-resistant individuals, beyond what is detectable in standard lipid panels, remains to be fully elucidated [87]. These considerations suggest that alternative or adjunctive therapies, such as GLP-1 receptor agonists or SGLT2 inhibitors, might be more appropriate initial choices in patients presenting with low HDL cholesterol and elevated ApoB.

The thiazolidinedione pioglitazone shows variable effects on ApoB and atherogenic lipoproteins, with the most significant benefits observed in patients with elevated triglycerides, metabolic syndrome, or nonalcoholic fatty liver disease. However, its clinical use must be balanced against potential adverse effects such as weight gain and fluid retention [88]. Biomarker-driven strategies, including ApoB or detailed lipoprotein particle profiling, may help identify patients most likely to benefit from pioglitazone, optimizing therapeutic decisions [89].

Taken together, these considerations highlight the need for further research on the role of ApoB as a routine lipid target in T2DM management, particularly in the context of emerging glucose-lowering therapies. Advanced lipoprotein testing methods, including nuclear magnetic resonance spectroscopy and ion mobility analysis, could improve detection of residual atherogenic risk beyond traditional lipid measures [90]. Moreover, the potential of combination therapies, such as GLP-1 receptor agonists with statins or PCSK9 inhibitors, to achieve greater reductions in ApoB and improve cardiovascular outcomes warrants further study. These questions underscore the importance of a personalized approach to reducing cardiovascular risk in patients with T2DM.

## Conclusion

ApoB has emerged as a central biomarker and therapeutic target in the prevention and management of ASCVD, particularly in individuals with T2DM. Unlike conventional lipid measures, ApoB provides a direct quantification of circulating atherogenic lipoprotein particles, enabling superior risk stratification and detection of residual cardiovascular risk, especially in settings where LDL-C levels are discordant with particle burden. The predictive accuracy of ApoB has been consistently validated across large-scale epidemiologic and interventional studies, demonstrating its utility both in untreated populations and among patients receiving lipid-lowering therapy.

In T2DM, ApoB levels are frequently elevated due to insulin resistance-driven dyslipidemia characterized by triglyceride-rich VLDL, small dense LDL, and reduced HDL-C. This pathophysiological profile underscores the need for biomarkers like ApoB that capture the true atherogenic particle burden, which may be underestimated by standard lipid panels. While some glucose-lowering agents, particularly GLP-1 receptor agonists, exert modest ApoB-lowering effects, the cardiovascular benefits of these therapies often extend beyond lipid modulation. Other agents, such as SGLT2 inhibitors and metformin, have minimal or inconsistent effects on ApoB but confer important cardiorenal protection through alternative mechanisms. Lipid-lowering therapies, especially statins and PCSK9 inhibitors, remain the most potent modifiers of ApoB and are essential components of comprehensive risk reduction strategies.

Future research should focus on integrating ApoB measurement into routine clinical practice and refining treatment algorithms to better identify and manage patients with elevated atherogenic risk. Personalized therapeutic strategies that incorporate ApoB levels, metabolic phenotypes, and advanced lipoprotein profiling offer significant potential to optimize treatment, particularly in complex populations such as individuals with type 2 diabetes mellitus. Embracing ApoB as a central component of cardiovascular risk assessment and therapeutic targeting may enhance the ability to reduce ASCVD burden and improve long-term outcomes across diverse patient populations.

## Figures and Tables

**Figure 1:**
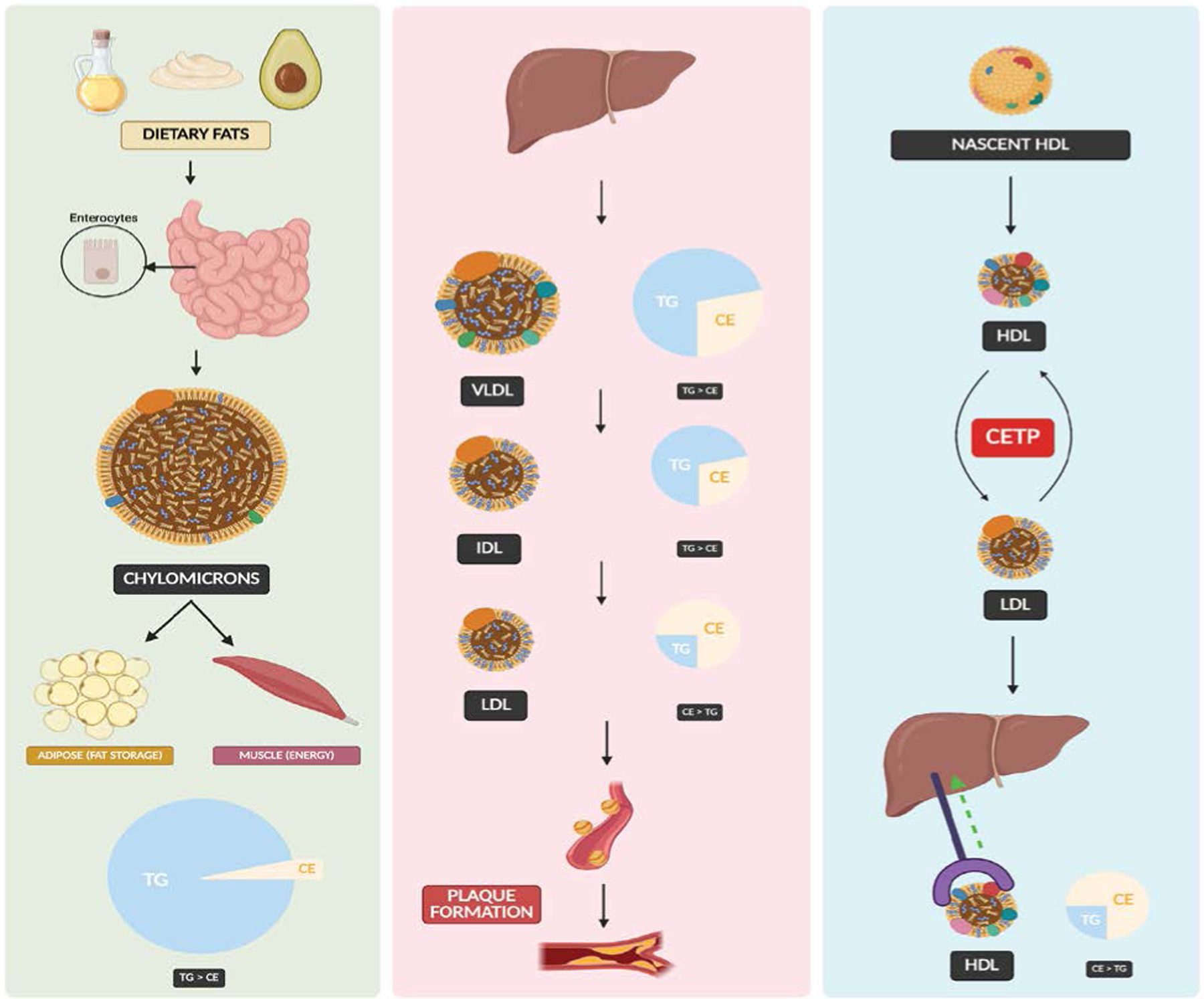
Left Panel: Dietary fats are absorbed in the small intestine and packaged into chylomicrons, which deliver triglycerides (TG) to adipose tissue for storage and to muscle for energy. Middle Panel: The liver synthesizes VLDL, which loses TG and is converted into IDL, and then LDL, contributing to cholesterol transport and potentially plaque formation. The pie charts illustrate changes in lipid composition (TG vs. CE) during lipoprotein transformation. Right Panel: HDL is synthesized as nascent particles and mature through reverse cholesterol transport, interacting with LDL via cholesteryl ester transfer protein (CETP), and returning cholesterol to the liver for excretion.

**Figure 2:**
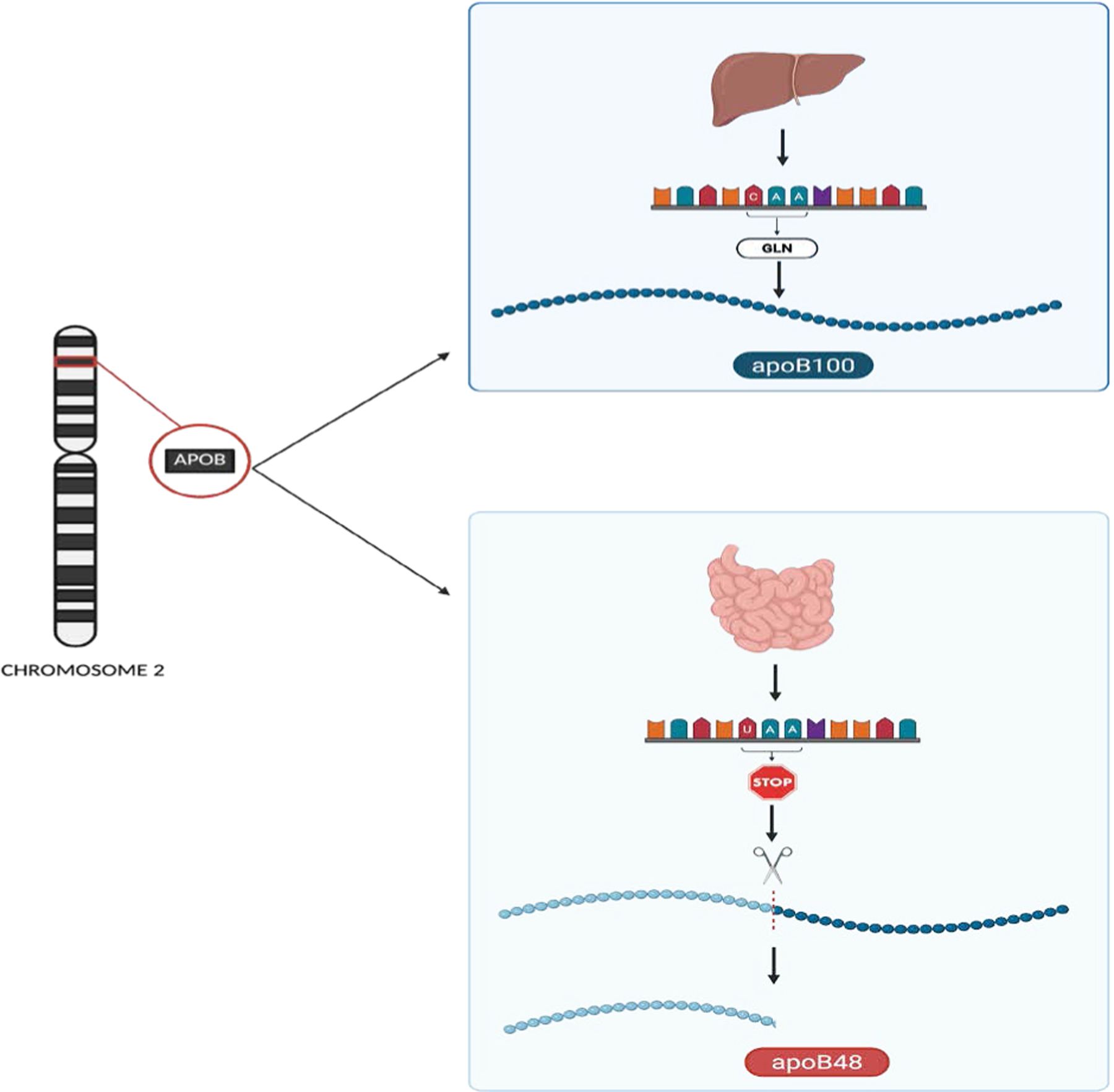
The *APOB* gene, located on chromosome 2, encodes Apolipoprotein B, which exists in two major isoforms depending on tissue-specific RNA editing. Top panel: In the liver, full-length translation of unedited mRNA produces ApoB100, which is essential for the assembly and secretion of VLDL. Bottom panel: In contrast, in the intestine, a cytidine deaminase enzyme complex edits a specific uridine in the mRNA, converting a glutamine codon (CAA) to a premature stop codon (UAA). The RNA editing results in translation termination and the production of ApoB48, a truncated protein required for chylomicron formation.

**Figure 3:**
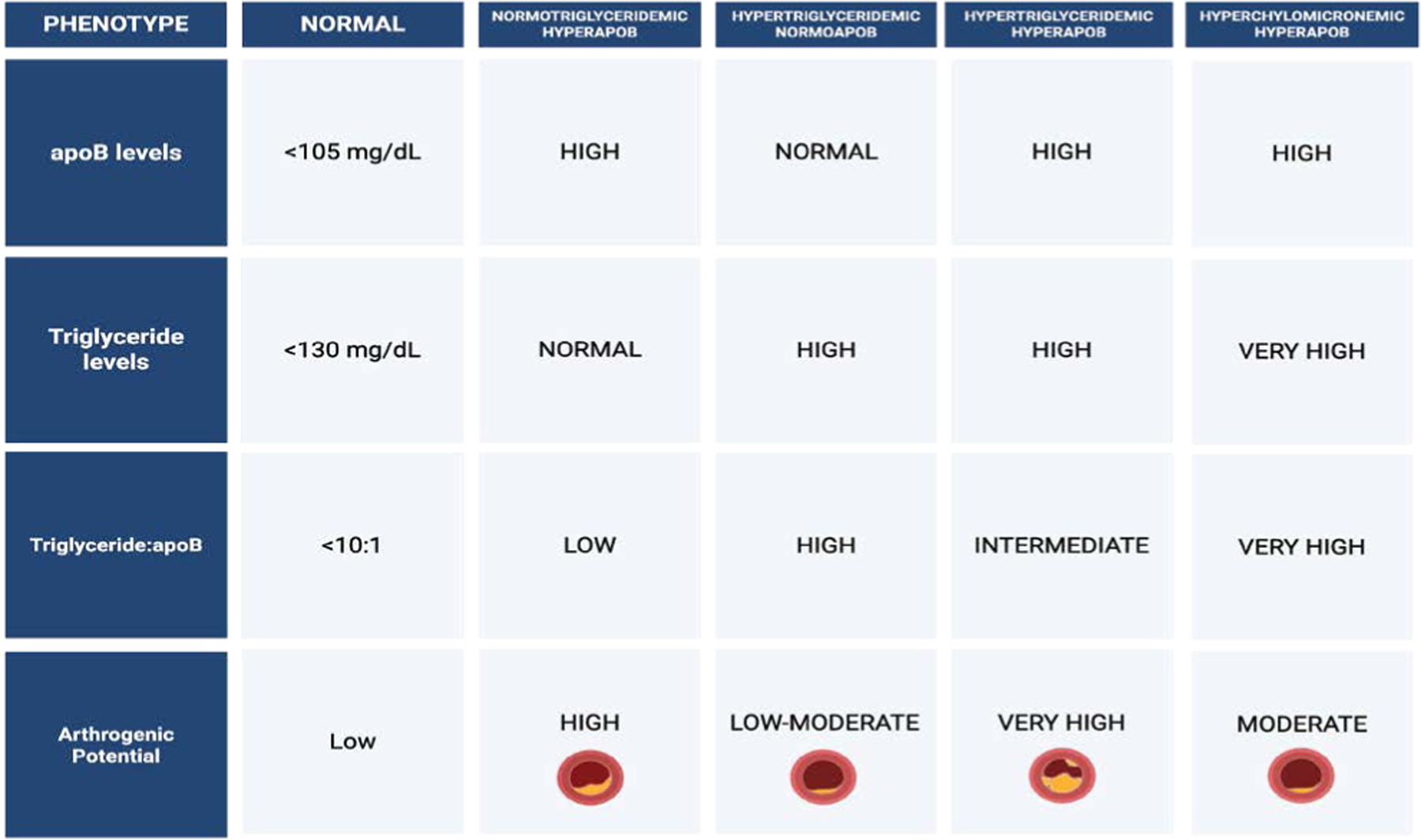
Lipid phenotypes characterized by ApoB and triglyceride profiles and their atherogenic potential. These profiles help stratify cardiovascular risk based on lipid particle composition rather than traditional lipid levels alone.

## Key Points

- Apolipoprotein (Apo) A is considered a good cholesterol due to its critical role in reverse cholesterol transport, whereas ApoB is atherogenic and is critically associated with the underlying pathogenesis of atherosclerosis and plaque formation.
- ApoB48 is essential in the assembly of chylomicrons that transport dietary triglycerides via the lymphatic system to peripheral tissues.
- Regulatory mechanisms involving microsomal transport protein and endoplasmic reticulum-associated degradation tightly control the availability and secretion of ApoB, linking nutrient status to lipoprotein metabolism and lipid homeostasis.
- Insulin resistance and associated metabolic disturbances drive a pathological increase in the density of ApoB particles.
- Genetic and epigenetic factors, and systemic inflammation can modify lipoprotein production and clearance and thus significantly influence the ApoB particle concentration.
- Four major dyslipoproteinemic phenotypes are defined by varying concentrations of ApoB, triglycerides, and changes in the composition of lipoprotein particles, reflecting distinct metabolic disturbances highlight the complexity of lipid metabolism and underscores the limitation of LDL-C as a solitary marker for cardiovascular risk.
- Recent clinical evidences strongly support ApoB as a more accurate and clinically relevant biomarker compared to LDL-C in the risk assessment, diagnosis, and treatment strategies for cardiometabolic diseases.
